# Geometric feature descriptor and dissimilarity-based registration of remotely sensed imagery

**DOI:** 10.1371/journal.pone.0200676

**Published:** 2018-07-19

**Authors:** Seyed M. M. Kahaki, Haslina Arshad, Md Jan Nordin, Waidah Ismail

**Affiliations:** 1 Center for Artificial Intelligence Technology, Faculty of Information Science and Technology, National University of Malaysia (UKM), Bangi, Selangor, Malaysia; 2 Faculty of Science and Technology, Universiti Sains Islam Malaysia, Bandar Baru Nilai, Nilai, Negeri Sembilan, Malaysia; IFRS, BRAZIL

## Abstract

Image registration of remotely sensed imagery is challenging, as complex deformations are common. Different deformations, such as affine and homogenous transformation, combined with multimodal data capturing can emerge in the data acquisition process. These effects, when combined, tend to compromise the performance of the currently available registration methods. A new image transform, known as geometric mean projection transform, is introduced in this work. As it is deformation invariant, it can be employed as a feature descriptor, whereby it analyzes the functions of all vertical and horizontal signals in local areas of the image. Moreover, an invariant feature correspondence method is proposed as a point matching algorithm, which incorporates new descriptor’s dissimilarity metric. Considering the image as a signal, the proposed approach utilizes a square Eigenvector correlation (SEC) based on the Eigenvector properties. In our experiments on standard test images sourced from “Featurespace” and “IKONOS” datasets, the proposed method achieved higher average accuracy relative to that obtained from other state of the art image registration techniques. The accuracy of the proposed method was assessed using six standard evaluation metrics. Furthermore, statistical analyses, including t-test and Friedman test, demonstrate that the method developed as a part of this study is superior to the existing methods.

## Introduction

Remote sensing is the method of obtaining information from objects or areas remotely, for example capturing earth imagery from aircraft, satellites or unmanned aerial vehicles (UAV) [1]. These images are usually captured by taking image sequences at different times or different viewpoints which needs to be registered or stitched together for further analysis. Image registration is the process of aligning two or more different images taken from a different time or different viewpoints. Registration of remotely sensed imagery is one of the basic processes in many aerospace applications, such as aerial reconnaissance and aerial mapping [2]. Some of the extant methods are based on rigid bodies of images that are utilized to extract transformation information for registration [3]. However, these methods cannot achieve appropriate results to register images with high deformations. Owing to these drawbacks, transformed images have been registered by using multi-resolution method [4]. In the multi-resolution method, the registration result at one resolution is used to estimate the parameters for other resolutions. However, the main drawback of these methods stems from the fact that all areas in digital imagery are considered similarly while changing the parameters in different resolutions. These weaknesses are considered in this study, the aim of which was to overcome the limitations. This is achieved by using a new method to extract the key points in different images, as well as applying an improved dissimilarity metric to find the correspondence points and estimate the transformation parameters.

Myronenko and Song [5] reformulated registration as a probability density estimation problem. While their method produces better results compared to other techniques, the method failed to register images with complex information and those affected by high transformation. These limitations are also addressed in the present study by using new dissimilarity metrics and correspondence ranking as a crucial component in image registration. Interest point detection and matching are essential aspects of image registration, as their aim is to extract the points conveying important information that are invariant to deformations.

Recently, corner points have been used in different application such as image retrieval, face recognition, and image registration. Current approaches based on this process can be divided into two main categories, referred to as single- and multi-scale detectors. Single-scale detectors cannot extract differently sized features in an image and are insufficiently robust under geometric transformation. To improve the performance of single-scale detectors, multi-scale versions have been introduced, which are more robust and can handle different signal effects. However, available multi-scale detectors are computationally expensive for more complex tasks compared to the single-scale counterparts. Thus, the need for a new technique that mitigates the existing detector challenges and achieves higher performance and lower localization error rates motivated us to develop an improved interest point detector and matching technique. The main contribution of this study thus stems from the development of an accurate feature correspondence method as an image registration technique based on a new dissimilarity metric and a novel approach for extracting the most accurate correspondence points using a line based feature extraction technique.

## Related works

The term ‘registration’ was introduced in the context of image processing by Becker [6], who patented a focusing camera that can be used to align two images [7]. The need for an imaging technology that can be applied to more complex imagery has motivated the researchers to develop faster approaches [8, 9]. Recently, several techniques using point matching by locating unique points in images have been proposed [10]. The analysis process applied to two or more images of a scene often depends on the ability to extract the correspondence features between specific sets of points in different images [7]. Wei, Han [1] proposed a small UAV based multi-temporal registration based on texture and geometric structure feature extraction and multi-feature guided point set registration to register aerial images. Thus, in this context, image registration refers to the process of extracting the correspondence points between two point sets in two or more different images [11]. In this work, the registration methods are employed when registering two images, with *I*_*r*_ denoting the reference image and *I*_*t*_ the target image. Hence, the correspondence problem can be defined as [12]:

$$I_{t}(x,y)=g(I_{r}(f(x,y))),$$ (1)

where *g* is the intensity or radiometric information and *f* is the coordinate transformation function, which matches two spatial coordinates (*x*,*y*) of the reference image to the spatial coordinates (*x*′,*y*′) in the target image, as shown below:

$$(x^{\prime },y^{\prime })=f(x,y).$$ (2)

Several authors have used this approach to match unique points in different images to improve the accuracy and speed of the existing techniques. The interest points in different images that are used for registration algorithms are called control points in this field. The aforementioned methods can be divided into intensity-based, frequency-based and feature-based registration approaches [13], all of which are described below.

### Intensity-based rgistration

In some registration methods, the intensity values sourced directly from the imagery can be compared without the need to select the interest points or control points. In these methods, the similarity value between the intensities in different images is used to determine their alignment. Similarity measurement methods, such as maximum likelihood [14] and mutual information [15], are typically employed to measure similarities between images. However, these approaches suffer from a high computational cost associated with measuring the similarity of entire images.

### Frequency domain registration

In this category, the goal is to find the best alignment between images based on the frequency domain information. The most common frequency domain registration is based on phase correlation, i.e., on the Fourier Transform. Scale and rotation invariant registration based on Fast Fourier Transform (FFT) method was proposed by Reddy and Chatterji [16]. Orchard [17] proposed an image alignment algorithm using the frequency domain, which minimizes the processing time required for searching the optimum global alignment and is invariant under linear intensity changes. Discrete Fourier (DF) registration method [18] is another recently developed technique that is based on nonlinear optimization combined with the matrix-multiply Discrete Fourier Transform. Thus, it produces a high accuracy image registration algorithm which is used as one of the comparison techniques for evaluation. There is a limited number of frequency-based approaches for developing registration algorithms, as these methods are not sufficiently efficient and are not robust under complex transformations, such as affine and projective.

### Feature-based registration

In this category of image registration techniques, the features called control points are extracted first, before finding the correspondence points. The algorithms in this category are faster and more reliable, as they utilize the points containing rich information. This category is further divided into two subcategories, pertaining to the methods based on either low-level or high-level features.

#### Registration based on high-level features

The methods in this subcategory allow the high-level features, such as regions, to be extracted, as well as to specify the objects (such as a building, a road or a river) in order to find the best alignment between the reference and the target images. The region can be matched based on the image area or the centroid points. Palenichka, Zaremba [19] developed an image registration technique based on finding and matching the objects common to both the reference and the target SAR imagery.

#### Registration based on low-level features

These techniques extract low-level features, such as edges, ridges or corner points of the reference and the target images before identifying the best-matched points based on the similarity measure techniques. Maximum likelihood estimation was used by Torr and Zisserman [20] in their image registration method. They used likelihood of the features rather than only counting the inliers which is the case used in original RANSAC. The contour matching approach was later proposed by Eugenio, Marqués [21], where it was applied to match the remote sensing imagery from single-sensor imagery with different viewpoints. Locally linear transformation (LLT) for both rigid and non-rigid remote sensing registration is proposed by Ma, Zhou [22] by creating a set of correspondence points and remove the outliers to estimate the transformation. Maximum likelihood estimation of a Bayesian model is formulated to determine the inliers and outliers. Their method is robust, scalable, and the complexity for rigid, affine and nonrigid shows a significant improvement, however, extracting SIFT features can make the process mathematically and computationally heavy especially in 3D space to calculate the gradient at specific pixels. Due to the wide variety of the image registration applications, several methods using different algorithms have been described in the literature. Despite their differences, most of the image registration problems are solved in the following three steps [23]:

- **Control point extraction:** A set of salient data, such as interest points or corner points, is selected automatically from the reference and target images.
  Contour-based corner detectors are the most commonly used detectors in the state of the art applications. Since Mokhtarian and Suomela [24] first introduced their curvature scale space (CSS)-based detector, many detectors in this class were developed by other researchers, who attempted to expand on their idea. Most of the enhancements to these detectors were aimed at improving the smoothing function. The enhanced curvature scale space (ECSS) detector [25] used a selected smoothing scale (σ) based on the curvature length (arc-length). This method, however, could not achieve similar repeatability in transformed images because the arc length of the curve may change under affine transformation. As this effect can significantly downgrade the detector performance, multi-scale curvature product was later proposed by Zhang, Lei [26] and He and Yung [27]. Their methods also provided a new version of the CSS-based method to improve the performance; however, they failed to overcome the aforementioned weaknesses.
  Corners are extracted as sub-pixel accuracy using Harris method by Torr and Zisserman [20] to find the correspondence points using an outlier removal approach. The subsequently developed chord-to-point distance accumulation (CPDA) [28] method was based on an affine-length parameter extracted from the curve, allowing the scale selection in the CSS methods to be improved. CPDA was intended to use the chord to point distance accumulation technique [29] to improve the localization of detected corners. However, as curvature points need to be calculated separately in the Gaussian scale, this method encounters difficulties when attempting to select the scale for different types of images. Another contour-based corner detector, known as anisotropic directional derivative (ANDD), was proposed [30] to remove the outliers using the ANDD classifier to extract the corner candidates. However, the ANDD implementation, which calculates ANDD for all the points at the contour, suffers from a high computational cost associated with the filter, causing the method to perform less optimally compared to the CPDA method.
  Yang, Pan [31] proposed a combination of different features and substituted into a mixture model to improve the registration accuracy and generality. In their approach, to calculate the similarity of geometric structure, the shape context, and the Euclidean distance are used. The scale-invariant feature transform (SIFT) is used to measure the scale space. This process can speed down the feature extraction process by calculating different features. In the control points detection method introduced in the present study, geometric mean projection transform and parabolic fit estimation are employed, thereby making the inclusion of the contour points that are located in the non-curve area redundant.
- **Control point matching:** Along with the control points extraction, control points matching is the main part of the registration algorithm. It necessitates definition of the correspondence relationship between sets of features. Several similarity measure metrics have been proposed in the literature, aiming to find the best matches between the feature sets in different images. The mathematical model of the matching criteria is given by [32]:
  $$\min_{T}F={\sum_{i}[I_{r}(T(x_{i},y_{i}))-I_{t}(x_{i},y_{i})]}^{2},$$ (3)
  where *I*_*r*_ and *I*_*t*_ represent the reference and target images, respectively, *T* denotes the transformation, *F* is the criteria function and *x*_*i*_,*y*_*i*_ are the coordinates of the points in the images.
  Geometric matching methods utilize spatial and geometric information pertaining to the points to detect the correspondence relationship in image pairs using the information related to these points, such as curvature, distance, and shape structure of the neighborhood information. Although the previous control point matching techniques, such as DT [33], ALTA [34] and DUTTA [35], approach the correspondence problem as a corner matching problem, each uses the features neighborhood information differently to yield the matching results. These methods are based on feature detection output information, which is insufficiently reliable for use in different correspondence applications, such as image registration.
  Vector field consensus (VFC) which is an alternative and improvement of the RANSAC method is proposed by Ma, Zhao [36] which tested on 2D and 3D datasets to handle a large number of outliers up to 90%. Their method starts with generating a set of initial correspondences and finding the final matched points by interpolating a vector field between two set of points which are generated using SIFT detector. They also proposed a nonrigid point matching [37] by minimizing the integrated square error or *L*_*2*_ estimator [38] to produce sparse and dense correspondences. Their method enables the process to deal with noise and outliers to handle the scale changes and rotation which shows the importance of the point matching process in registration applications.
- **Transformation estimation and reconstructing the target image:** In this step, the transformation parameters are estimated using the feature matching results. The transformation matrix based on homography transformation can be extracted using geometric information of the correspondences. In the last step, the target image is transformed into the original image using the transformation matrix. This can be accomplished by employing interpolation techniques or optimize the energy function [31].

## Image registration method

This section presents the proposed image registration method, based on the geometric mean projection transform, square Eigenvector correlation, and correspondence ranking. A new robust feature matching technique is employed to extract the most similar features based on their neighbourhood intensity and line features, allowing the image registration application to produce more accurate results. The first image is referred to as the reference image, while the second one is denoted as the target or sensed image. In this process, the reference image remains unchanged, while the target image is transferred using transformation matrix, allowing it to take new coordinates.

**Fig 1:**
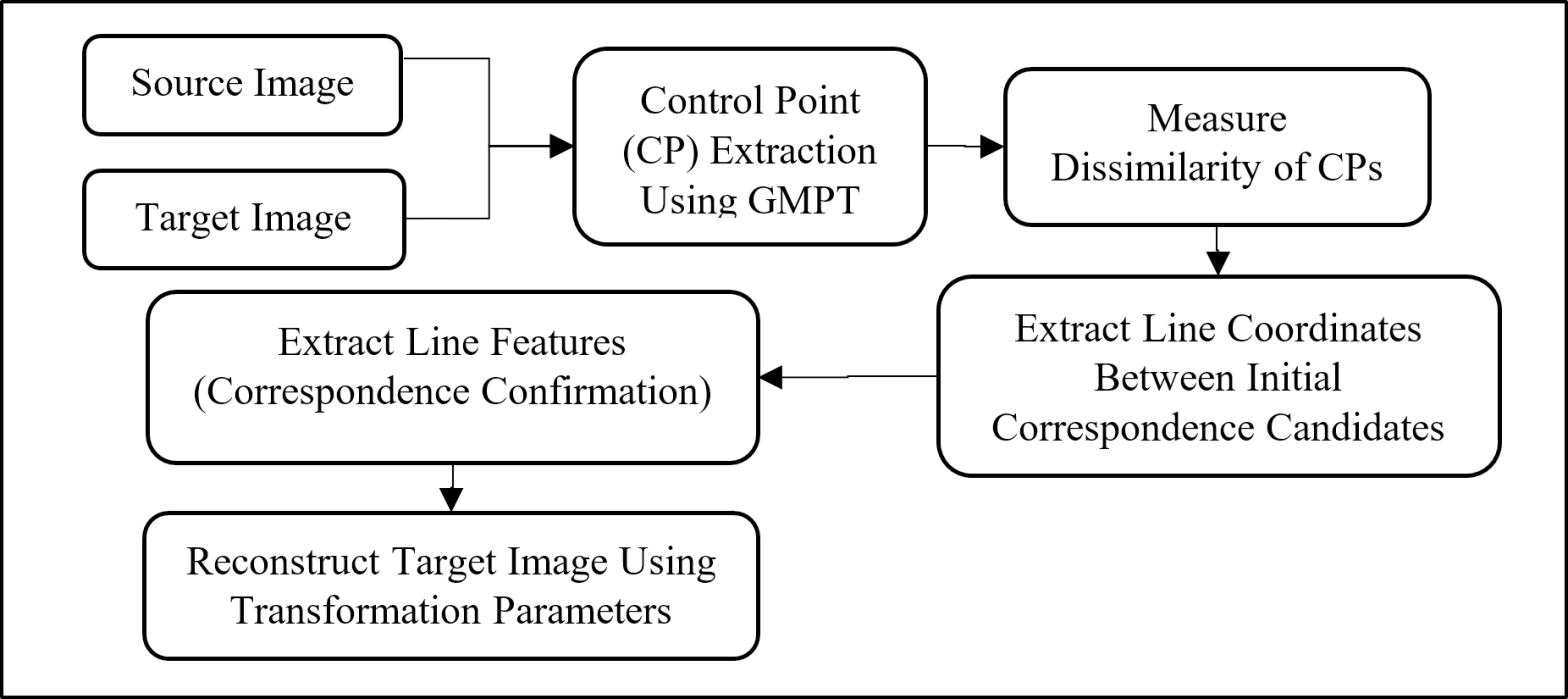
The stages of the image registration framework.

Control points in the images are used to find the correspondence points in both the reference and the target images. The features are extracted in the first stage by using projection-based transform method. Then, a new dissimilarity metric and dissimilarity matrix of the best-fit candidates are extracted to find the most similar features. Finally, the transformation parameters, referred to the homogeneous matrix, are estimated to extract the transformation between image pairs. In the final phase, the goal is to transform the target image coordinates to those of the reference points, in order to correctly register the images. Achieving this objective requires an efficient homogeneous matrix estimation and image transformation using statistical solutions. These stages are presented in Fig 1 as the image registration framework.

Many authors have adopted the affine transformation in their feature matching approaches [39]. However, in the real world, some images cannot be matched using affine transformation. In recognition of this issue, in this study, homography transformation is considered. To obtain the homography parameters, at least four sparse feature correspondences must be used to calculate the homogeneous matrix [40].

The transformation and matching problem is formulated by considering *I*_*r*_ as the reference image and *I*_*t*_ as the target image. Let us denote a set of feature vectors with *l* number of features derived from the reference image *I*_*r*_ by $P={\left\{p_{i}\right\}}_{i=1}^{l}$ and that from the target image *I*_*t*_ with *m* number of features by $Q={\left\{q_{i}\right\}}_{i=1}^{m}$. The transformation can be then annotated as *T* defined as *T*:*P* → *Q*, which represents the correspondence relation of all the points from *P* to *Q*.

According to the transformation theory, a transformation from *P* to *Q*, whereby a feature set ${\left\{p_{i}\right\}}_{i=1}^{l}$ is mapped to ${\left\{q_{i}\right\}}_{i=1}^{m}$ over the same field, is a function *F*:*P* → *Q* and is referred to as the transformation function. In geometry, a homography transformation includes translation, scaling, homothety, similarity transformation, rotation, reflection, shear mapping and compositions, as well as any combination of these and their sequences. Homography transformation of *F*:*P* → *Q* can be expressed as {*Q*} = *M*{*P*} ⊕ {*v*},*P* → *MQ* + *v*, where *M* is a linear transformation and *v* is a vector on *Q* and *p*(*x*,*y*) ∈ *P* and *q*(*x*,*y*) ∈ *Q*. To satisfy homography transformation with unknown variables, at least four coordinates in P and Q are needed, where *p*_*i*_ ≠ *q*_*j*_, in order to obtain a unique registration *F* that satisfies the condition *Q* = *F*(*P*) [40].

### Geometric mean projection transform

Feature extraction is an essential process in many image processing and computer vision applications, such as location recognition [41], vision-based recognition and mining [42], image registration, etc. A projection based transform which incorporating the line rotation signal acquisition is considered in this study to extract feature points. This method belongs to the contour-based detectors class, in which the candidates are extracted using the contour information. Its results are used to extract the final feature points via an approximation based on a parabolic fit. The proposed method is developed in three main steps. In the first step, the edge map is extracted using Canny edge detector, and the omitted edge pixels are filled using the edge recovery method. Next, feature candidates are selected from the edge map of the image using a projection transform. In the final step, the corner points are identified using an approximation of the parabolic fit.

Edge detection is the first step performed in the contour-based corner detection methods. Available methods such as Canny can fail to detect the edge pixels in some parts of the image, especially in curved areas. As a result, some of the feature points may not be detected due to edge missing. In the present study, these points are recovered using a gap removal technique. In this technique, information on the neighbourhood from the center point of a mask is considered in each part of the image that contains the edge information. In the first step, all empty pixels located inside the window contains an edge area are selected as the potential candidates. These candidates can be selected as a gap if the window includes an edge and some of its pixel coordinates lies at a straight line equation. If the candidate and its neighbouring edge pixels create two crossover lines, it is used to complete the edge map. These steps are repeated sequentially until all the missed edge points are identified, and the map is completed. This approach ensures that the method recovers all the omitted points which can reduce the occurrence of false-negative points.

While previous CSS-based detectors employ Gaussian smoothing to ensure that the image is differentiable at the aliased edges and the features, they also increase the false-positive rate in the aliasing edges [43]. Candidate selection using the geometric mean projection transform (GMPT) method can mitigate this problem and thus significantly reduce the false-positive rate as well.

**Fig 2:**
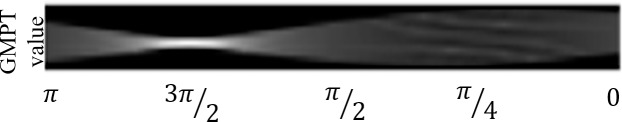
The GMPT result for a straight line of an image contour using Eq (4) in different directions, where *θ* ∈ [0,*π*]. Lighter regions indicate greater function values and black denotes zero.

GMPT is a transform that consists of the integrals over straight lines (L) of the foreground in a digital image in different directions (*θ*). If *f*(*X*) =*f*(*x*,*y*) is a function of the image line signals (L) in $R^{2}$, then GMPT is a transform of L, where the geometric mean of the integrals in vertical and horizontal directions considering *S*(*X*) as the standard deviation of *f*(*X*) is calculated using Eq (4):

$$GMPT(L)=\prod_{i=1}^{n}{\left(\int_{Lx}S(X)|dX|,\int_{Ly}S(Y)|dY|\right)}_{\theta \in [0,\infty )},$$ (4)

where GMPT calculates the mean of the integrals in an input image in both vertical and horizontal directions of line L and *S*(*X*) is the standard deviation of *f*(*X*). The GMPT parameters can detect the available angular contours from a straight contour on the edge map of the objects in an image. Fig 2 illustrates the mean projection on the straight line in an image contour.

**Fig 3:**
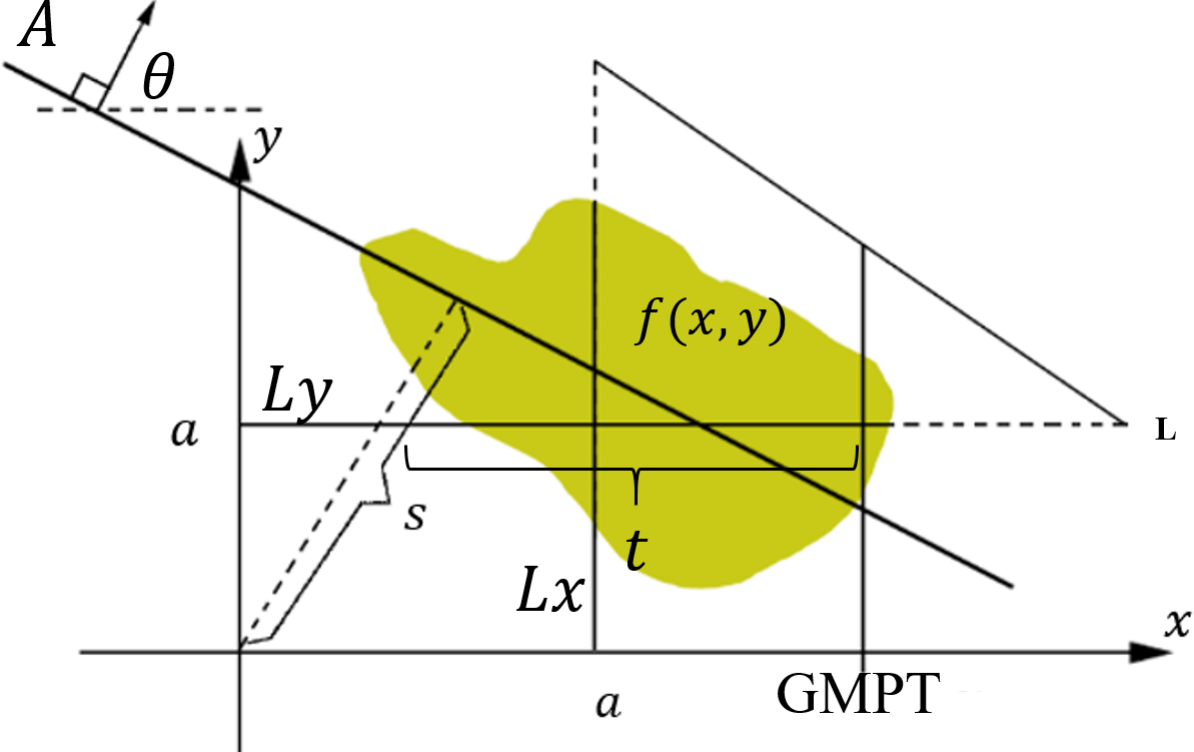
Definition of GMPT transform for a function of the image foreground f(x,y). The GMPT value for direction *θ*, at the location *a* is presented as *GMPT*(*a*,*θ*).

Fig 3 illustrates the definition of *GMPT* for each straight line *L* of the object. For each x and y coordinate, represented by *a*, there is a vertical and horizontal value; thus, the GMPT value for *GMPT*(*a*,*θ*) is the mean of the integrals. Mean integral descriptor is the result of the *GMPT* that calculates the mean of all integral values over a straight line in different directions. As both horizontal and vertical directions are considered in the *GMPT* representation, it yields more detailed information on the objects with fewer required rotations compared to other projection based methods, such as Radon transform.

Considering the arc-length t on the line *L*, the Eq (4) can be written as:

$$\left(x(t),y(t)\right)=\frac{1}{2}\left(\left(\left(t\sin(\theta )+s\cos(\theta ),(-t\cos(\theta )+s\sin(\theta \right)\right)•\left(\left(-t\cos(\theta )+s\sin(\theta ),(t\sin(\theta )+s\cos(\theta \right)\right)\right)$$ (5)

where *s* is the Euclidean distance from the origin to L, *θ* is the vector angle, L is in the Cartesian coordinate system, and (*θ*,*s*) are the transform parameters on $R^{2}$ for all lines.

The image rotation is computationally expensive for methods based on image rotation, such as Radon transform, Trace transform and GMPT. Moreover, image rotation changes the intensity and location of the image pixels, potentially introducing errors into transformations [44]; therefore, a non-rotational projection is employed. The advantage of this method is its ability to capture all the image information without the need for any image rotation. This is achieved by capturing the information from four sides of an image. In the projection technique, unlike other transforms, such as Radon and Trace transforms, the capturing function is not limited to a specific function. From each projector location, all the lines in [0,π4] radian are used to calculate and capture the image information. These four projectors move through the range to cover all available line directions. For each projector, the coordinates for each radian in θ∈[0,π4] are calculated based on the image size.

**Fig 4:**
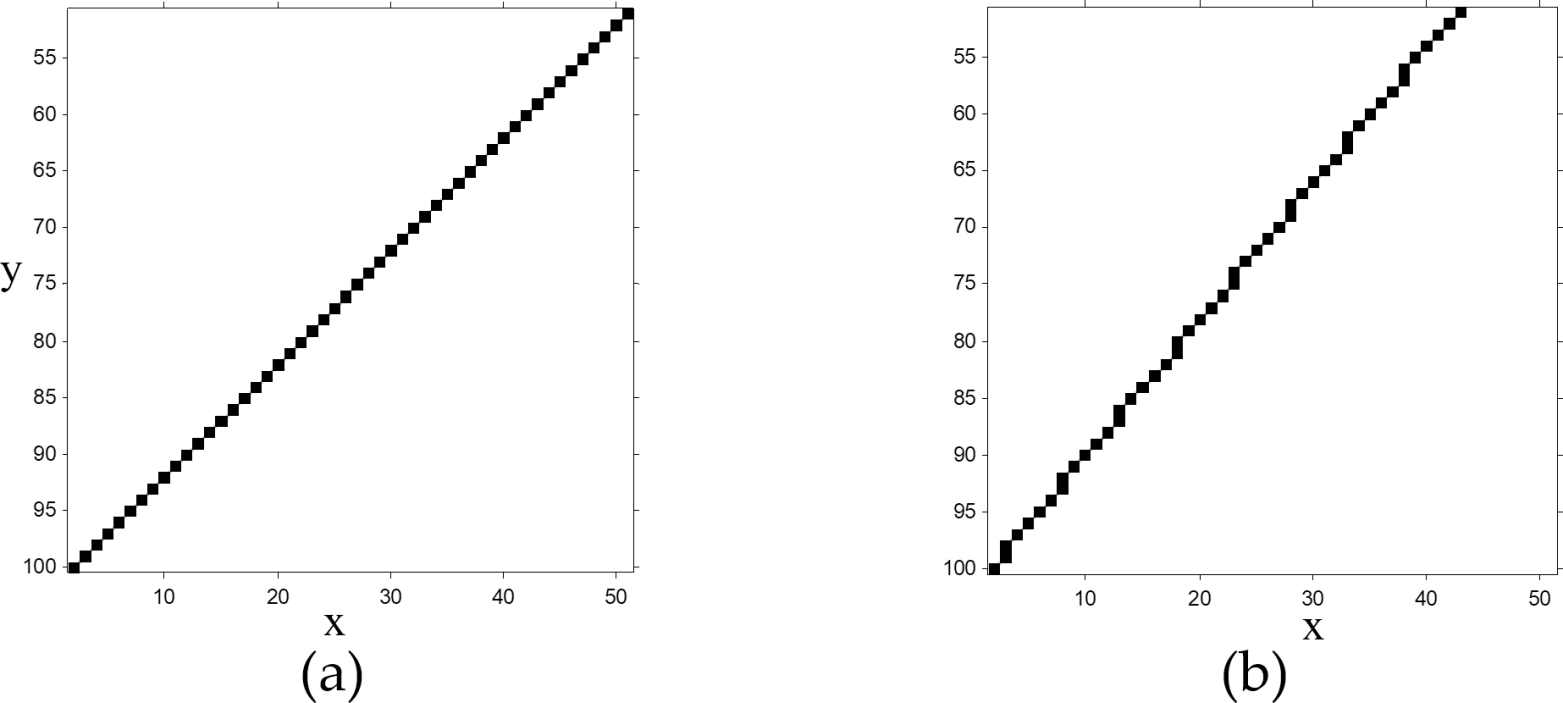
A sample of *f*(*x*) results for (a) θ=π4 and (b) θ=π3.

As the most straightforward calculation based on the Cartesian coordinate system, the process starts with the calculation of the start and endpoint coordinates of the fourth projector to capture the image information. Given the straight-line equation, the pixel coordinates of the line for θ∈[0,π4] that belongs to the capturing information from the fourth projector are obtained based on the line equations. The sampled line pixels for capturing the image information are depicted in Fig 4. Using all the available adjacent lines pertaining to the four projections, all the pixels of an image can be captured without any image rotation.

Once the candidates have been extracted using GMPT, the exacted corner coordinates in the selected contour area must be identified. An approximation of the polygonal fit is used to find the final control point coordinates. Considering two candidate points *p*_*i*_ and *p*_*j*_, orthogonal lines meet at the center point of the parabola ρ. Hence, if *p*_*i*_ denotes *ρ*(*p*_*i*_,*ε*), *p*_*i*_*p*_*j*_ is a segment *δh*(*p*_*i*_*p*_*j*_) ≤ ε, whereas if *p*_*i*_*p*_*j*_ intersect at *d*_*i*_ + 1,…,*d*_*i*_ − 1, the parabola radius passing the points is given by:

r=[1+(dydx)2]32|d2ydx2|, (6)

where *dx* and *dy* are extracted using the *p*_*i*_ to *p*_*j*_ points inside the parabola area. Additionally, the method is adjustable for detecting the low- and high-order corners in different image scaling by adjusting the *ϑ* value as the focal control parameter. Based on the experimental results, we defined the general expression for *ε* as:

$$\varepsilon >\frac{w\times 2}{\vartheta },$$ (7)

where ϑ is the focal control parameter, and *w* is the moving window width. The truth condition in Eq (7) guarantees that the ε value does not exceed the curve radius. *ϑ* and *w* are input arguments that are adjustable by the user to support high scaling images and are defined for an image with 515 × 512 pixels as 9 and 5, respectively, by default. The effect of changing these parameters is discussed in the evaluation section.

### Squared eigenvector correlation

This section focuses on feature matching technique, which is based on the proposed dissimilarity metric to improve the correspondence performance. In image processing applications, such as image registration, the detected interest points need to be matched in the reference and target images in order to obtain the final results. In this section, a new geometric point matching technique capable of finding the correspondence points in both the reference and the target point sets is obtained. To describe the method, correspondence problems are formulated before discussing the solutions.

Matching all the points in $P={\left\{p_{i}\right\}}_{i=1}^{l}$ to $Q={\left\{q_{i}\right\}}_{i=1}^{m}$ using homography invariant transformation *T*:*P* → *Q* can be achieved using four points selected from P and Q that exhibit the greatest accuracy among all the features in the set. Let us assume that the vector set $\vec{V}={\left\{{\vec{v}}_{i}\right\}}_{i=1}^{l}$ represents the neighborhood information of the input features, i.e., ${\vec{v}}_{1}\in [\delta <p_{1}<\delta ],$ where *δ* is the scale of the neighborhood information of the first feature *p*_1_ that satisfies the condition *p*_1_ ∈ *P*. Then, the best correspondence set is the one with the maximum similarity (*S*), for which the probability (*ρ*) of correspondence to another feature is the greatest.

While we observed that *δ* = 7 is the optimum value to achieve high performance registration results, the effects of different *δ* values on the registration results are discussed further in the evaluation section. Referring to the earlier case of *P* with *l* number of features and *Q* with *m* number of correspondence features, a general similarity index can be defined as:

$$\vec{PQ}({\left\{p_{i}\right\}}_{i=1}^{l},{\left\{q_{i}\right\}}_{i=1}^{m})=\sum_{i=1}^{l\times m}\left|p_{i}-q_{i}\right|,$$ (8)

where *l* is the size of *P* and m is the size of *Q*.

As similarity metrics are the core of many image processing and signal processing algorithms, improving them can significantly and positively affect the final application performance. In the extant literature, the term ‘dissimilarity’ is often used interchangeably with similarity distance *d* that, when applied to a set of pixel values *S*, can be defined as $d:S\times S\to R^{+}\cup \{0\}.$ Some of its main properties are:

- Self-similarity: For all *W* ∈ *S*,*d*(*W*,*W*) = 0.
- Positivity: For all *W* ≠ *T* ∈ *S*,*d*(*W*,*T*) > 0.
- Symmetry: For all *W*,*T* ∈ *S*,*d*(*W*,*T*) = *d*(*T*,*W*).
- If *d*(*W*,*T*) = 0 and *d*(*T*,*Q*) = 0 = > *d*(*W*,*Q*) = 0.

Based on the comprehensive review of the extant literature related to similarity metrics, eleven similarity distances deemed to exhibit superior performance compared to others are considered in this study, namely sum of absolute differences, locally scaled sum of absolute differences, zero-mean sum of absolute differences, squared sum of intensity differences, zero-mean sum of squared differences, locally scaled sum of squared differences, normalized cross-correlation, zero-mean normalized cross-correlation, relative entropy, structural similarity and normalized mutual information.

To evaluate these similarity distances, one hundred features are extracted and their neighbour information considered. These features from standard datasets are classified and marked as either similar or non-similar manually. The results of this process indicate that, even when the threshold value is optimally chosen, it is not sufficient to produce an accurate and reliable similarity score. To overcome this issue and improve the performance, a new dissimilarity metric is introduced in this section.

To ensure that a feature is invariant, invariant feature vector similarity is required. Based on empirical evidence, Von Neumann entropy *S*(*ρ*) is a good candidate, as it is invariant under changes. In other words, *S*(*ρ*) = *S*(*UρU*^†^) with *U* unitary transformation, as the entropy depends on the eigenvalues of the density matrix only, and can be defined as [45], [46]:

$$S(\rho )=-Tr\rho \;\ln\;\rho =-\sum_{1}^{N}\lambda_{i}\;\ln\;\lambda_{i},$$ (9)

where *λ*_*i*_ are the eigenvalues of *ρ* and N is the number of elements in *ρ*. For each vector ${\vec{v}}_{i}$ of the feature *i* in both *P* and *Q*, the entropy is calculated and inserted in the probability accumulative matrix *p* which is l × m matrix given below.

$$p={\left[\begin{array}{ccc} {D_{Ent}}_{1,1} & \ldots & {D_{Ent}}_{1,m}\\ \vdots & \ddots & \\ {D_{Ent}}_{l,1} & & {D_{Ent}}_{l,m} \end{array}\right]}_{l\times m},$$ (10)

where *D*_*Ent*_ is the distance entropies between each two features, which are calculated using Eq (11) [45]:

$$SEC(P_{l},Q_{m})=\sqrt{{S(\rho_{l})}^{2}-{S(\rho_{m})}^{2}/\bar{K}}.$$ (11)

Row *l* and column *m* in matrix *p* show the dissimilarity between feature *p*_*l*_ and *q*_*m*_, which should be minimal, while $\bar{K}$ is the mean of *ρ*_*l*_ and *ρ*_*m*_. If dissimilarities in row *l* are minimal, the minimum *D*_*Ent*_ column indicates the maximum similarity of the features in the feature sets *P* and *Q*. Thus, the similarity matrix *S*(*P*,*Q*) provides the ranking of the similarity indices for features.

### Correspondence ranking and registration

Matching the features allows the transformation information to be extracted, which is the key requirement for registration problems and is widely used in remote sensing, computer vision and medical imaging [47]. For this goal, the most similar features from reference and target are considered to estimate the transformation parameters. To find the best correspondence features to estimate the transformation parameters, correspondence ranking is required. For this goal, considering the initial correspondences are available, line coordinates between features are extracted. Then, the line features including the frequency and intensity variation are considered to compare and rank the correspondences. However, intensity information is used to register images with similar spectral contents, additional features (including color histogram, frequency, and hue) are employed to achieve more accurate matching process.

Given two sets of features *P* and Q from the reference and target images, respectively, several imaginary domain lines can be considered between these features. These lines are denoted as $\hat{L}$ with the length of *δ*. For example, ${\hat{L}}_{v_{t},u_{t}}$ is the line between features *u* and *v* from the target image with a line length of $\delta_{v_{t},u_{t}}$. The line $\hat{L}$ includes *δ* pixels as $\hat{L}={\left\{l_{i}\right\}}_{i=1}^{\delta }$ where each *l*_*i*_ includes three feature values denoted as *F*,*h* and *H*, respectively denoting frequency, hue and histogram value of the line.

Let two points *p*(*x*,*y*) and *p*′(*x*′,*y*′) be the input of the line coordinate extractor. Then, the coordinates of the points passing by the line between *p* and *p*′ are the output produced. Given that the equation describing a straight line is typically written as *y* = *mx* + *b*, where *m* is the gradient or the slope of the line and *b* is the y-intercept, the gradient is calculated first using Eq (12) in order to obtain the equation of the line passing through *p* and *p*′.

$$G=\frac{\Delta y}{\Delta x},\theta \neq 90{}^{\circ},$$ (12)

where Δ*x* and Δ*y* are the changes in *x* and *y*, respectively. To calculate the y-intercept, *p* or *p*′ can be substituted in the line equation:

$$b=p(y)-\left(\frac{\Delta y}{\Delta x}\times p(x)\right),$$ (13)

**Fig 5:**
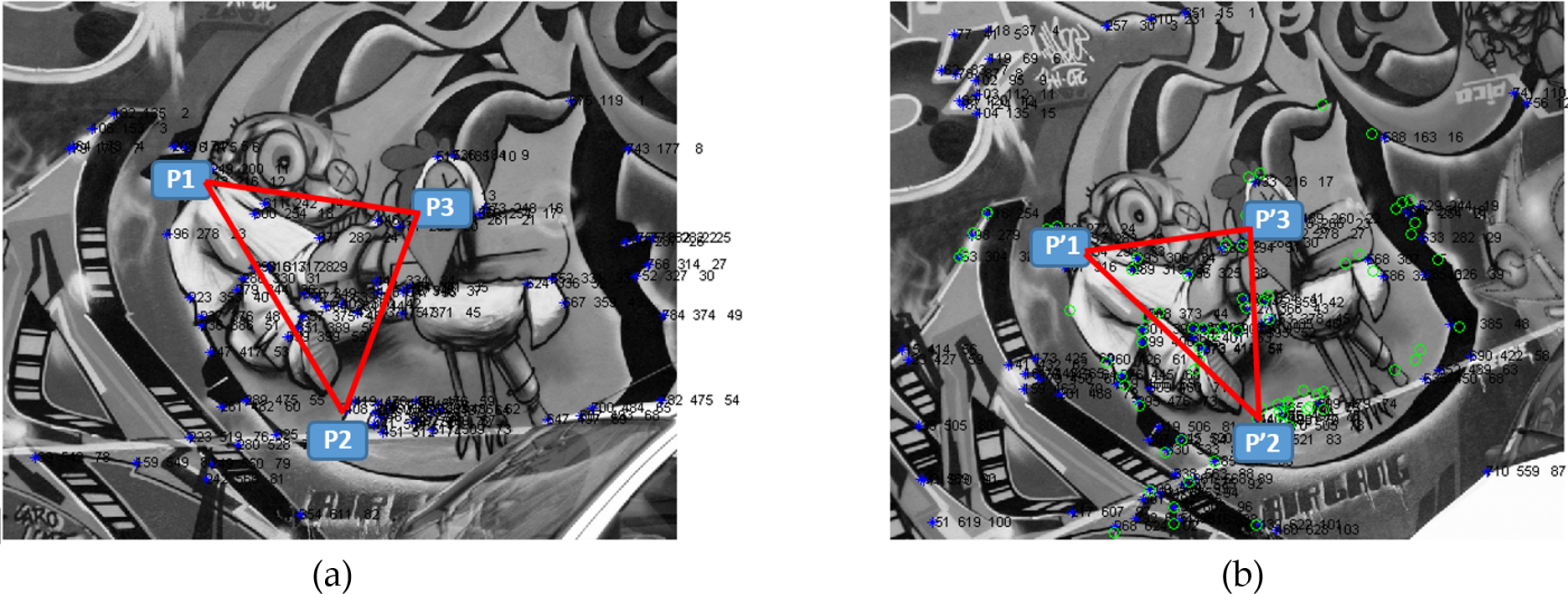
The line coordinates (the red lines) used to extract the features. (a) Original Graffiti image. (b) The transformed image.

The remaining coordinates can be easily calculated using the above line equation passing through two points *p* and *p*′. Given that *θ* = tan^−1^*G* and *G* = *undeffined* for *θ* = 90°, to calculate the coordinates in this case, all *y* input points with the same *x* axis are considered as the output of the line coordinate extractor. Similarly, for *θ* = 0°, all the *x* inputs with the same *y* axis are considered as the outputs. Fig 5 presents the original Graffiti image (left) and its transformed image (right) including three selected points and the lines between them. *H* = [7.6,-2.9,2.2;3.34,1.0,-7.6;3.4,-1.4,1.0] is a high-level transformation between these images which makes the matching and registration more difficult.

Considering that images are 2D representations of reality, and the function *f*(*x*,*y*) is defined in spatial coordinates (*x*,*y*) in an image plane, each function in the image describes how the colors of the images in different color maps vary in the space. Alternative image representation is based on spatial frequencies of color, intensity or edge variations over the image pixels. This triple representation using a spectrum of different frequency components is completely equivalent to the conventional spatial representation. Thus, the direct conversion of a 2D spatial function *f*(*x*,*y*) into the 2D spectrum *F*(*u*,*v*) of spatial frequencies is used to extract the features of the lines.

**Fig 6:**
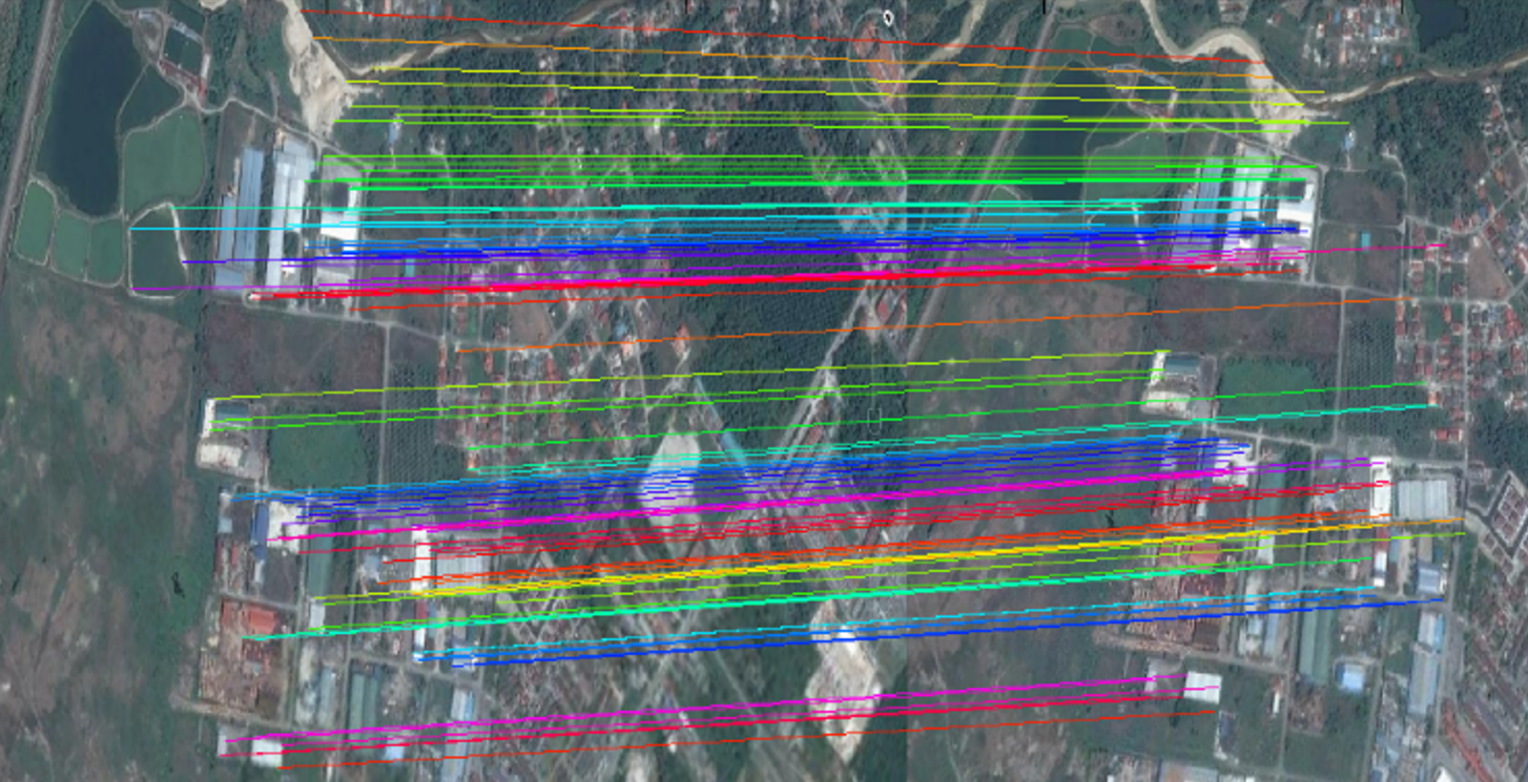
Matching and registration result of an aerial image. a) matching results which the correspondences are confirmed using the best-fit candidate matrix and the correspondence ranking, b) overlapped image after registration, and c) the difference after the registration which shows the error in white pixels.

After extracting the line coordinates, as shown in Fig 5, the pixel values of the points in the image through which the line is passing are considered to extract the line features. The width of the extracted line between every two points can be adjusted, depending on the application requirements. In the Graffiti image used as an example here, the use of the three points is shown in Fig 5, allowing 286 pixels to be extracted between the points p1 and p2, to producing the first line. The best-fit candidate and correspondence ranking create the final set of the correspondence points that can be used to estimate the transformation parameters based on the comparison of the line features between the points. An aerial image and its transformation are examined the matching and registration process, and the results are shown in Fig 6. The aerial input image (Fig 6(A)–left) and the transformed image (Fig 6(A)–right) which includes viewpoint change (angle of tilts *θ* = 24°) shows that the matching technique and registration process can achieve good results in highly deformed aerial imagery.

In order to obtain the homogeneous matrix H, which can be utilized to transform all other points from the reference to the target images, the transformation parameters need to be estimated. Then, these parameters are used to reshape the target image to the reference image. As the projective transformation projects every image into its protectively equivalent image, provided that it is projective invariant [40], the transformations (projective, affine, translation, rotation, etc.) of the reference image to the target image can be accomplished. The goal here is to find the transformation matrix H that can map all the points from the reference image to the target image.

A projective transformation is a linear transformation of homogeneous H that provides (*x*,*y*) ↔ (*x*′,*y*′) mapping. Projective transformation is also referred to as homography or collineation transformation, and is defined by Eq (14) [40].

$$\left(\begin{array}{c} x^{\prime }\\ y^{\prime }\\ 1 \end{array}\right)=\left[\begin{array}{ccc} h_{11} & h_{12} & h_{13}\\ h_{21} & h_{22} & h_{23}\\ h_{31} & h_{32} & h_{33} \end{array}\right]\times \left(\begin{array}{c} x\\ y\\ 1 \end{array}\right),{\left(x,y\right)}^{\prime }=H(x,y),$$ (14)

where H is a 3×3 homogeneous matrix. In this most general transformation between the real-world and image plane under imaging with a perspective camera, obtaining the 3×3 matrix is essential for establishing the transformation properties. If the number of points n > 4, the H can be determined uniquely [40]. Moreover, as the overall scale of H is arbitrary, the *h*_33_ can be set to 1. Using the four points required to estimate the H matrix, Eq (15) can be defined as:

$$\begin{array}{c} x_{i}{}^{\prime }\left(h_{31}x_{i}+h_{32}y_{i}+1\right)=h_{11}x_{i}+h_{12}y_{i}+h_{13}\\ y_{i}{}^{\prime }\left(h_{31}x_{i}+h_{32}y_{i}+1\right)=h_{21}x_{i}+h_{22}y_{i}+h_{23} \end{array}\to \left(\begin{array}{ccccccccc} x_{i} & y_{i} & 1 & 0 & 0 & 0 & -x_{i}{}^{\prime }x_{i} & -x_{i}{}^{\prime }y_{i} & -x_{i}{}^{\prime }\\ 0 & 0 & 0 & x_{i} & y_{i} & 1 & -y_{i}{}^{\prime }x_{i} & -y_{i}{}^{\prime }y_{i} & -y_{i}{}^{\prime } \end{array}\right)h=0.$$ (15)

where h = (*h*_11_,*h*_12_,*h*_13_,*h*_21_,*h*_22_,*h*_23_,*h*_31_,*h*_32_,1)^*T*^ is the matrix H written in the vector form. With respect to the four points. The above equation can be presented in the *Ah* = 0 form, where *A* is an 8×9 matrix. Using *n* > 4 points, the transformation matrix H can be calculated using Eq (15).

Using the dissimilarity matrix and the best-fit matrix, the transformation parameters *h*_11_,*h*_12_,*h*_13_,*h*_21_,*h*_22_,*h*_23_,*h*_31_,*h*_32_ can be estimated using Eq (15). These parameters are sufficient for mapping all the control points from the reference image to the target image. In this stage, using homogeneous matrix, all the feature point coordinates (control points) of the reference image are mapped to the target image, as presented in Fig 6. These correspondence points are sufficient to register the target image to the reference image efficiently and with high accuracy.

**Fig 7:**
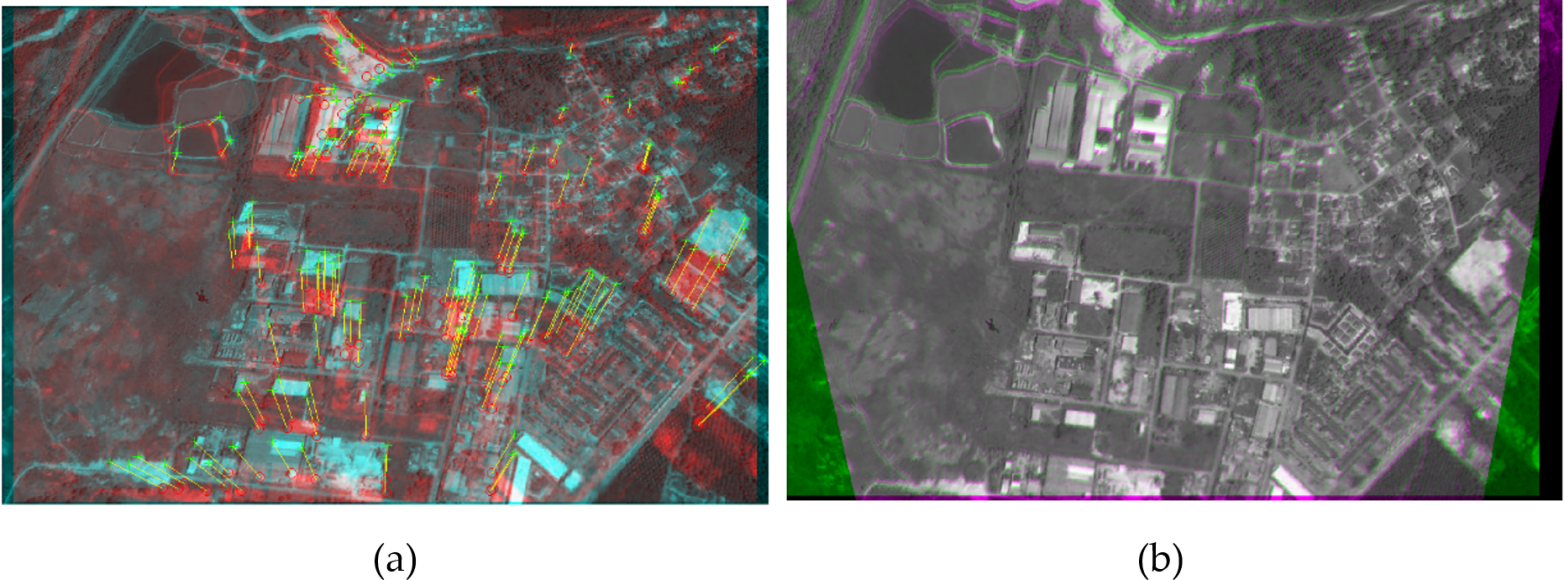
The registration process of remote sensing imagery. (a) Matching the points from the reference to the target image. (b) Final registration result.

In the final step, the target image is transformed into the reference image. Any pixel of the target image can be represented as the form of the pixel coordinates. These coordinates need to be transformed into an inverse homogeneous transform. The homogeneous transformation matrix was estimated in the previous section and can be used here to transform the points from the reference to the target image, as shown in Fig 6. Using all the pixel points in the reference image $P={\left\{p_{i}\right\}}_{i=1}^{l}$ and the target image $Q={\left\{q_{i}\right\}}_{i=1}^{m}$, a transformation from the former to the latter can be expressed as *T*:*P* → Q. Similarly, the transformation from the target to the reference image is the inverse of that process and is denoted as *T*^−1^:Q → P. Using the transformation parameters estimated in the preceding sections, the inverse transform can be estimated [48]. The inverse transformation can be calculated using the properties of the transformation matrices. The inverse transformation matrix converts all the pixel coordinates of the target image to the new coordinates that represent their true positions. This process is the final step of the image registration application and is shown in Fig 7.

The overlapped reference and target images, including feature movement, are presented in Fig 7(A) to show the matching result of high-resolution remote sensing imagery before the registration process. The matching result is used to define the homogeneous parameters and register the target image to the reference image with a high accuracy, as presented in Fig 7(B). The grey section in Fig 7(B) presents the common parts in reference and target images after the registration process.

## Evaluation

### Visual experiments

**Fig 8:**
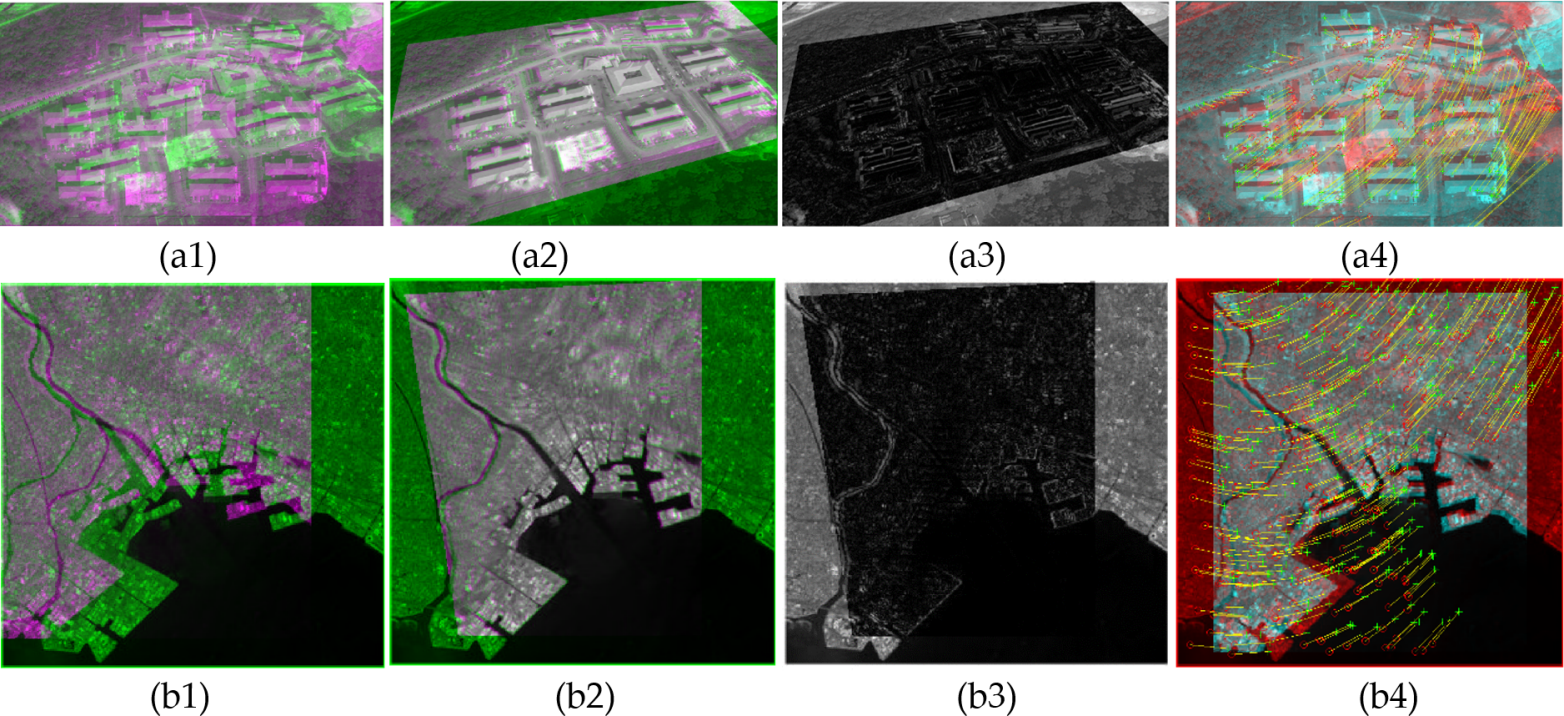
Image registration results. (a1-a3) A sample of two satellite images for registration, (a1) overlapped images after the registration, (a2) the difference after the registration, and (a3) feature matching results. (b1-b3) A sample of two images for registration (form UCSB-IKONOS dataset), (b1) overlapped images after the registration, (b2) the difference after the registration, and (b3) feature matching results.

This section presents the results obtained by applying the image registration technique developed as a part of this study. The reference and the target images are considered as the input of the registration method, while the output is the recovered target image, which should be as much as possible similar to the reference image. To measure the performance of the method, we need to examine the similarity between the recovered image and the reference image. The robustness of the proposed techniques was measured using the “Featurespace” and “IKONOS” datasets [49]. The Featurespace dataset is sourced from the University of Oxford [50] which includes eight different set of images with different transformation properties purposely developed for feature matching evaluations. The IKONOS dataset includes panchromatic imagery with a spectral range of 760–850 μm which is a fine resolution craft operated by GeoEye. To visually present the registration performance using satellite imagery, the images sourced from the aforementioned standard datasets were considered in the evaluation process. Fig 8 illustrates the matching and registration results pertaining to the aerial images in different viewpoints.

Fig 8A1 and 8B1 present the overlapped reference and target images after the registration of satellite images. The difference image presented in Fig 8A2 and 8B2 is the error resulting from the registration of the target image into the reference image. In the difference image, brighter pixels indicate points that are not registered correctly, which are denoted as errors, while the darker pixels indicate points that are registered with higher accuracy. The matching result, including the movement of the points during the registration process provided in Fig 8A3 and 8B3, indicates the movement of features during the registration process which shows that most of the correspondence points are detected correctly.

### Evaluation metrics

Different evaluation methods can be used to measure the performance of the image registration such as image quality assessments. The process includes measuring the quality of the reconstructed or registered image relative to the reference image. Peak signal-to-noise ratio (PSNR) and structural similarity (SSIM) are the most common to measure the performance by comparing the original image and the reconstructed target image [51]. PSNR is an engineering term which can be measured based on the mean square error to compare the similarity of two input image or signals. SSIM is also a method for measuring the similarity between two input images which is designed to improve on other methods such as PSNR and MSE which are not consistent with human eye perception. In the present study, six different image quality assessment techniques were considered. The accuracy of the proposed method was assessed using PSNR, SSIM, Laplacian mean-square-error (LMSE), normalized absolute error (NAE), normalized cross-correlation (CC) and average differences (AD). The definition of these evaluation metrics is given in Table 1.

**Table 1:** Image quality assessment techniques.

Quality measure technique	Definition
Peak to signal ratio (PSNR)	$PSNR=10{\times log}_{10}\left(\frac{c_{max}^{2}}{\frac{\sum_{i=0}^{m-1}\sum_{j=0}^{n-1}{\left(R(i,j)-T(i,j)\right)}^{2}}{mn}}\right)$ $C_{max}\leq \left\{ \begin{array}{l} \;1,\;in\;double-precision\;intensity\;images\\ 255,in\;8-bit\;unsigned-integer\;intensity\;images \end{array}\right.$
Structural similarity (SSIM)	$SSIM(x,y)=\frac{4\mu_{x}\mu_{y}\sigma_{xy}}{\left({\mu_{x}}^{2}{{+\mu }_{y}}^{2}){{(\sigma }_{x}}^{2}{{+\sigma }_{y}}^{2}\right)}$ $\sigma_{x}=\sqrt[{2}]{\frac{1}{N-1}\sum_{i=1}^{N}{\left(x_{i}-\mu_{x}\right)}^{2}}$, $\sigma_{xy}=\frac{1}{N-1}\sum_{i=1}^{N}\left(x_{i}-\mu_{x})(y_{i}-\mu_{y}\right)$, $\mu_{x,y}=\frac{1}{N}\sum_{i=1}^{N}{x,y}_{i}$
Laplacian mean-square-error (LMSE)	$LMSE=\frac{\sum_{i=1}^{m}\sum_{j=1}^{n}\left[\omega (x_{i,j})-\omega ({x^{\prime }}_{i,j}\right)]}{\sum_{i=1}^{m}\sum_{j=1}^{n}{\omega (x_{i,j})}^{2}},$ $\omega (x_{i,j})=x_{i+1,j}+x_{i-1,j}+x_{i,j+1}+x_{i,j-1}-4x_{i,j}$
Normalized absolute error (NAE)	$NAE=\frac{\sum_{i=1}^{m}\sum_{j=1}^{n}\left|x_{i,j}-{x^{\prime }}_{i,j}\right|}{\sum_{i=1}^{m}\sum_{j=1}^{n}\left|x_{i,j}\right|}$
Normalized cross-correlation (NCC)	$NCC=\frac{\sum_{i=1}^{m}\sum_{j=1}^{n}x_{i,j}.{x^{\prime }}_{i,j}}{\sum_{i=1}^{m}\sum_{j=1}^{n}x_{i,j}^{2}}$
Average differences (AD)	$AD\frac{\sum_{i=1}^{m}\sum_{j=1}^{n}\left(x_{i,j}-{x^{\prime }}_{i,j}\right)}{m.n}$

where *x* and *y* are the coordinates of the image pixels, *m* and *n* are the dimensions of the source image, *R*_*xy*_ is the source image, and *T*_*xy*_ is the target image.

These standard evaluation techniques are employed to compare the proposed method with other well-known techniques, namely multimodal and dimension registration (MMD) [52], multi-modality registration (MM) [53], maximum likelihood consensus random sample consensus (MAC-RANSAC) [20], evolutionary strategy (ES) [54], discrete Fourier (DF) [18], coherent point drift (CPD) [5], and Vector field consensus (VFC) [36]. Some of these techniques such as CPD, VFC, and MAC-RANSAC are point matching techniques which does not calculates the transformed images. To evaluate these methods and compare with others, the result of the point matching has been used to estimate the transformations and the target image is transformed using standard MATLAB transformation called imwarp. Finally, to evaluate the robustness and accuracy of the proposed technique relative to other approaches, two statistical analysis tests are applied to the results, namely t-test and Friedman test. As will be discussed later, the results of these analyses indicate that the proposed method outperforms the other methods.

### Evaluation results

As previously noted, the proposed method was compared with some other related techniques, all of which were evaluated using several standard techniques and different sets of images. The first test was performed on an IKONOS image under rotation. Firstly, the effects of $\frac{\pi }{36}$ radian rotation were considered for all the methods and the results obtained are presented in Table 2.

**Table 2:** The results of applying different registration techniques on IKONOS image.

Method	AD	LMSE	NAE	NCC	PSNR	SSIM
multi modal and dimension (MMD)	0.000124	0.000268	0.018415	0.998401	83.84534	0.987845
multi-modality (MM)	0.005723	0.005583	0.064809	0.982463	70.66224	0.867134
maximum likelihood consensus random sample consensus (M-RANSAC)	0.000559	0.00034	0.018208	0.99754	82.81904	0.989538
evolutionary strategy (ES)	0.055593	0.045814	0.258453	0.857367	61.52081	0.48169
discrete Fourier (DF)	0.074636	0.069925	0.378503	0.793324	59.68451	0.292849
coherent point drift (CPD)	0.073471	0.066575	0.354935	0.800711	59.89766	0.277848
Vector field consensus (VFC)	0.000532	0.000046	0.013005	0.990983	84.04614	0.985325
Proposed	6.01E-05	0.000262	0.012368	0.999316	84.14568	0.989842

PSNR, NCC, and SSIM are similarity scores of the reconstructed target image and the reference image. Higher values of these measures indicate higher accuracy while LMSE, AD, and NAE indicate the system error. The results yielded by the different methods under the rotation effect presented in Table 2 confirm that the proposed method has both a lower error rate and a higher similarity in most of the cases compared to the other techniques. VFC and M-RANSAC generates accurate feature correspondence set and shows better results based on LMSE while they outperformed by the proposed method based on other metrics. The SSIM of these two methods are also very close to the proposed method which indicates these methods produced promising results.

**Fig 9:**
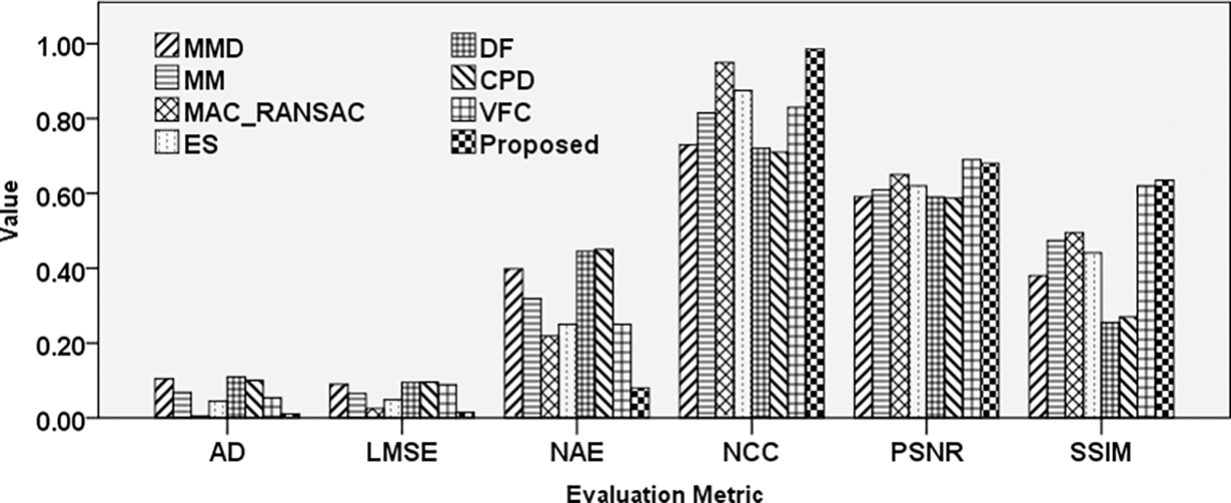
Comparison results of different registration techniques applied to LANDSAT image affected by an affine transformation.

The affine transformation is a significant effect in image registration applications and was thus considered in recently developed registration methods [11]. Fig 9 illustrates the comparison of different techniques under affine transform using different evaluation metrics.

The proposed technique exhibits higher performance in most of the cases relative to other approaches. The VFC method achieved slightly higher PSNR rate and MAC-RANSAC achieve slightly lower absolute difference error. These can be the result of the deformation of the reconstructed image after inverse affine transform. In general, the most accurate result was achieved by the proposed method, followed by MAC_RANSAC, VFC, and ES.

A combination of affine and rotation applied to AVIRIS images and the results are presented in Table 3. These results indicate that the proposed method outperforms other techniques, using most of the evaluation methods. Registration result based on NAE shows that the VFC has slightly higher performance compare to others. Comparison using different evaluation metrics helps to evaluate different methods more precisely to be able to rank the methods more accurately. However, SSIM and PSNR are the most accurate and reliable measurement techniques which can be used as the basic metrics. The best LMSE is achieved by ES method and VFC achieved the best NAE score.

**Table 3:** Results of different registration techniques applied to AVIRIS image affected by an affine transformation.

Method	AD	LMSE	NAE	NCC	PSNR	SSIM
**MMD**	8.36E-02	0.072637	0.345744	0.773838	59.51920	0.366823
**MM**	0.019474	0.031367	0.207278	0.921783	63.16608	0.499695
**M-RANSAC**	0.073829	0.065888	0.319675	0.796927	59.94273	0.464026
**ES**	0.015000	0.021246	0.183768	0.943597	64.85796	0.520260
**DF**	7.46E-02	0.069925	0.378503	0.793324	59.68451	0.292849
**CPD**	0.062487	0.066004	0.358212	0.806308	59.93513	0.294148
**VFC**	0.033829	0.041890	0.127643	0.901927	61.82310	0.521340
**Proposed**	0.011007	0.034139	0.138747	0.961275	65.79824	0.560363

**Fig 10:**
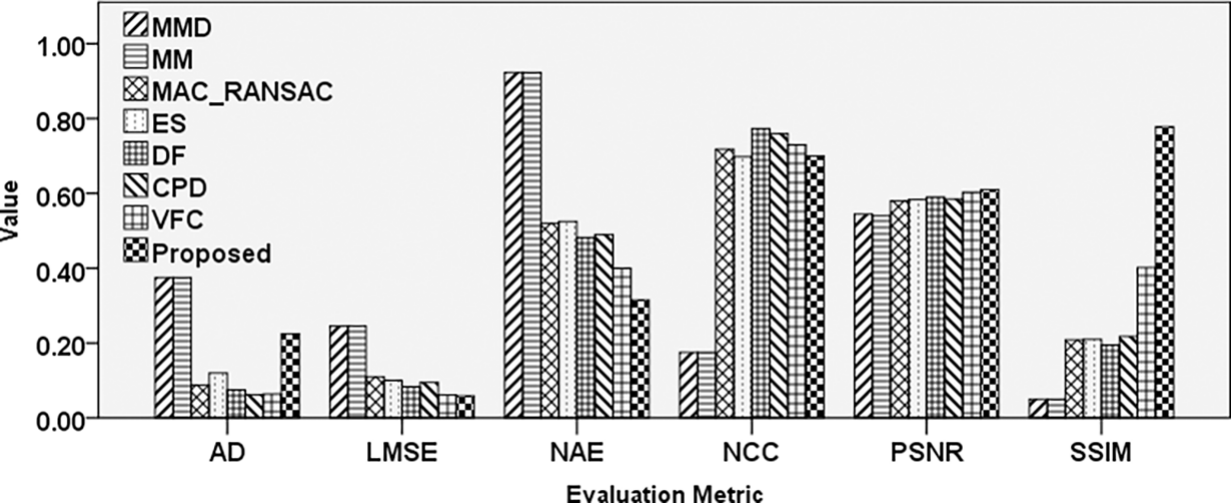
Comparison results of different registration techniques applied to an aerial image affected by mirror transformation.

The comparison results pertaining to the performance of various techniques regarding the ability to register the mirror image are illustrated in Fig 10.

Evaluation of different techniques under mirror effect indicates higher NCC for DF, CPD, and VFC methods compared to the proposed method. Lower AD error achieved by MAC-RANSAC, ES, DF, CPD, and VFC techniques, followed by the proposed method. However, the results pertaining to PSNR and SSIM, which are the most accurate image assessment techniques, indicate that the proposed method exhibits superior performance under this effect.

Graffiti is an image sourced from the Featurespace dataset, which was used because it provided different viewpoints of the scene. Table 4 presents the results of applying different techniques to these images.

**Table 4:** Results obtained by applying different registration techniques on Graffiti images sourced from the Featurespace dataset.

Method	AD	LMSE	NAE	NCC	PSNR	SSIM
**MMD**	0.382466	0.221274	0.969024	0.061579	54.68151	0.003713
**MM**	0.382466	0.221274	0.969024	0.061579	54.68151	0.003713
**M-RANSAC**	0.044264	0.082309	0.508167	0.750605	58.97634	0.180398
**ES**	0.295730	0.194481	0.885530	0.210587	55.24203	0.018362
**DF**	0.006710	0.099585	0.592057	0.798761	58.14887	0.083446
**CPD**	0.010769	0.100993	0.584870	0.762540	58.08790	0.073260
**VFC**	0.032065	0.081212	0.433142	0.823452	57.99354	0.198765
**Proposed**	0.020017	0.082765	0.405140	0.832844	58.95233	0.206769

**Fig 11:**
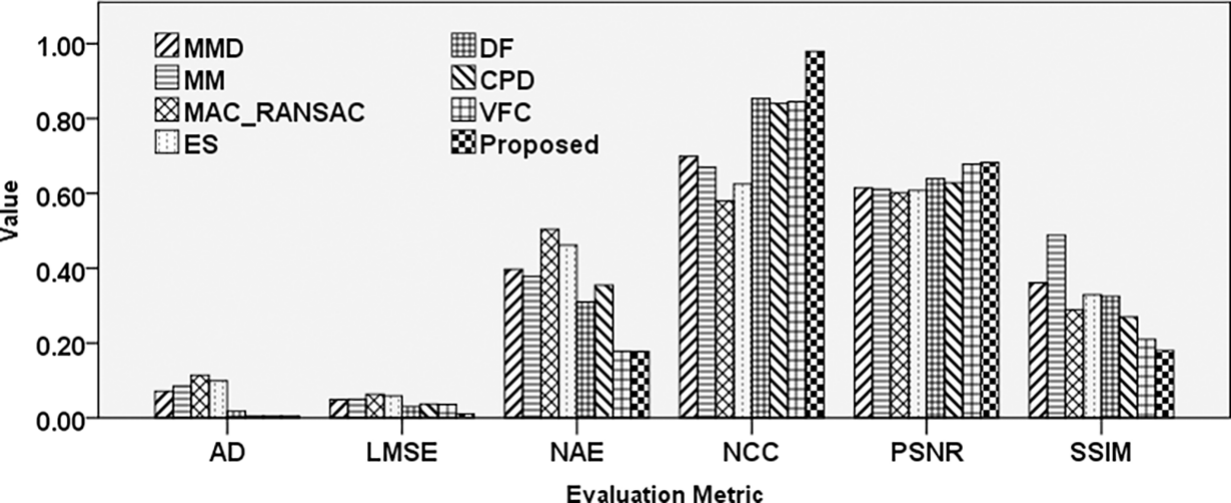
Comparison results of different registration techniques applied to aerial images.

The results presented in Table 4 indicate that the MAC_RANSAC method is superior to the proposed approach, based on LMSE and PSNR. The minimum AD error was achieved by the ES method, while the proposed method was superior in terms of the NCC, NAE, and SSIM. Fig 11 illustrates the results obtained by registering aerial images using different techniques.

The results presented in Fig 11 indicate that the proposed method achieved the lowest error rate, followed by the VFC, CPD, and DF techniques. Maximum SSIM value was achieved by the MM method, while the proposed method yielded maximum PSNR and NCC, followed by DF, VFC, and CPD techniques.

### Parameter effect

Table 5 presents the effects of parameter selection on the registration results. For each set of parameters, PSNR and SSIM values, as the most accurate comparison measures, were calculated based on the registered images as well as the response time in seconds. Three main parameters, namely the focal control (ϑ), moving window size for the corner classifier (w) and scale of neighbourhood information (*δ*) of dissimilarity measurement in corner matching, were considered. The results indicate that the *δ* size has a greater effect on PSNR and SSIM compared to others because it directly affects the matching results. Focal control parameter and moving window size influence the corner detection results and the number of detected feature points.

**Table 5:** Effects of different parameter changes.

	**ϑ = 3, w = 5, δ = 7**	**ϑ = 5, w = 5, δ = 7**	**ϑ = 7, w = 5, δ = 7**	**ϑ = 9, w = 5, δ = 7**	**ϑ = 11, w = 5, δ = 7**
**PSNR**	69.89545	68.62192	71.25489	72.91976	70.21568
**SSIM**	0.659875	0.601254	0.645896	0.664191	0.665126
**Time**	4.2548	3.7598	3.01548	2.1458	1.9854
	**w = 3×3, ϑ = 9, δ = 7**	**w = 5×5, ϑ = 9, δ = 7**	**w = 7×7, ϑ = 9, δ = 7**	**w = 9×9, ϑ = 9, δ = 7**	**w = 11×11, ϑ = 9, δ = 7**
**PSNR**	72.88268	72.91976	71.25489	71.35987	70.21459
**SSIM**	0.663256	0.664191	0.661254	0.659875	0.654589
**Time**	1.7086	2.1458	3.5985	4.1458	5.5896
	**δ = 3, ϑ = 9,w = 5**	**δ = 5, ϑ = 9,w = 5**	**δ = 7, ϑ = 9,w = 5**	**δ = 9, ϑ = 9,w = 5**	**δ = 11, ϑ = 9,w = 5**
**PSNR**	56.25489	60.98245	72.91976	72.92458	73.45874
**SSIM**	0.454589	0.512569	0.664191	0.664862	0.669864
**Time**	1.0015	1.8548	2.1458	3.5865	4.9864

We observed that the PSNR and SSIM values increased with the increase in *δ* because its greater value results in the correlation window considering more information when calculating the dissimilarity values. However, this change is not significant for *δ* ≥9, while a higher *δ* value increases the response time significantly. When the value of *w* was increased, the robustness of the registration increased slightly, since accurate features were detected with higher *w*. This effect is not significant because the weak features are ignored in the correspondence ranking process. The performance also slightly increased with the increase in ϑ. Selecting higher ϑ and *w* values resulted in a slight increase in the response time. The optimum result based on PSNR, SSIM and response time was thus achieved with ϑ = 7, *w* = 5 and *δ* = 7.

### Statistical analyses

In previous sections, several evaluations of the proposed technique and its comparison with other well-known approaches were performed and discussed. Different datasets were employed in order to assess the performance of these techniques using standard evaluation methods. To demonstrate that the differences between the results obtained are statistically significant, a t-test was performed. This was followed by the Friedman test, in order to rank the techniques based on their performance. The findings of these two statistical tests confirm that the difference between the results obtained by the proposed method and those used in the evaluations are statistically significant.

T-test assesses if the averages or means of two groups are reliably different from each other. While examining the means may reveal a difference, that is insufficient to demonstrate that the difference is reliable. In other words, the t-test is an inferential statistic that does not merely describe the sample data, but also allows the findings to be generalized to the entire population beyond the sample under the test. To achieve this goal, the t-test can be performed to analyze the differences in the data between and among the groups. Thus, as the comparison is always performed between two sets of results, the results of the proposed method are subjected to comparisons with each remaining technique individually and the p-values are presented in Table 6. The use of p-values in statistical hypothesis testing is common in many fields of research which is the probability for a given statistical model that, when the null hypothesis is true, the statistical summary (such as the sample mean difference between two compared groups) would be the same as or more extreme than the actual observed results. To ensure that the t-test has the sufficient statistical power to detect the difference present in the data, the evaluation results yielded by the registration process of 32 independent data samples were utilized. These data samples roughly followed a normal distribution. Moreover, each group had a similar number of independent data points, thus avoiding inaccuracies that could be introduced by comparing large to small groups. The results of the pairwise comparisons are presented in Table 6.

**Table 6:** The t-test results for the pairwise comparison of the proposed method with each of the remaining techniques.

Method	p-value
**Proposed-MMD**	0.036352
**Proposed-MM**	0.029814
**Proposed-ES**	0.046464
**Proposed-MAC-RANSAC**	0.040820
**Proposed-DF**	0.041199
**Proposed-CPD**	0.044808
**Proposed-VFC**	0.049321

The results presented in Table 6 confirm that the differences between the results of the proposed technique and those obtained by other techniques are statistically significant, as the p-values are below 0.05. The higher p-values correspond to greater similarity in performance and vice versa. Therefore, among the comparison techniques, the performance of the VFC and ES approaches are the most similar to that of the proposed method, while that of the MM method is the most dissimilar.

The ranking of the techniques using the Friedman test is presented in Table 7. Friedman test is a non-parametric statistical test commonly used to detect significant differences between sets of data. Although the Friedman test ranks each method with respect to various measurement techniques, given the importance and power of the PSNR, in the present study, it was adopted as the primary measurement point in the Friedman test for all aforementioned evaluation tests. In the first step, the null hypothesis was defined as “there are no significant differences in the performance of different methods” and the alpha value was set as 0.05. In this test, the degrees of freedom was set to 6, which was calculated using *df* = *k* – 1, where *k* represents the number of groups. The results of the Friedman test indicated that the null hypothesis should be rejected, as they confirmed that the performance of the proposed technique is superior to that of the other methods based on the mean rank.

**Table 7:** The Friedman test results for different image registration techniques.

*Method*	*Mean*	*Std. Dev.*	*Min/Max*	*Mean Rank*
**MMD**	64.7065	9.57595	54.25/83.85	3.38
**MM**	63.7844	7.15122	54.25/76.55	3.63
**MAC-RANSAC**	65.4496	8.08709	57.89/82.82	4.38
**ES**	61.9814	4.31222	55.24/70.54	2.75
**DF**	62.1063	3.46555	58.15/67.88	3.63
**CPD**	62.2533	4.03247	58.09/70.55	3.63
**VFC**	66.3421	8.24321	55.15/78.34	4.54
**Proposed**	70.3454	8.53942	58.95/84.15	6.63

The asymptotic significance *p* = 0.009 and Chi-Squared = 64.789 was achieved for N = 32 data samples using df = 6 for seven different groups. In the present study, a cutoff value of 0.05 was considered in order to reject the null hypothesis and confirm that the difference between the sets of data is statistically significant. *N* shows the number of data points used in the Friedman test, and *df* denotes the degrees of freedom. Finally, “Chi-Squared” is the distribution of a sum of the squares of *k* independent standard normal random variables. The Friedman test results confirm that the highest rank is assigned to the proposed method.

### Response time

To measure the speed of different methods, their average response time in seconds was measured, and the results are presented in Table 8. All methods were executed in Matlab and performed on a PC with an Intel (R), Core (TM) i5-4570, CPU at 3.20 GHz and 6GB of RAM. The response time comparison was fair, as it was conducted under identical conditions using the same system. The results indicated that, except MMD and MM methods, the proposed method is faster than other techniques. However, the proposed method achieved higher accuracy compared to both MMD and MM.

**Table 8:** Average response time for different image registration techniques.

*Method*	*MMD*	*MM*	*MAC-RANSAC*	*ES*	*DF*	*CPD*	*VFC*	*Proposed*
Average Res. Time (Sec.)	1.2565	1.9654	3.4869	2.3256	2.2145	4.1565	3.83022	2.0125

## Conclusion

In this paper, a new image registration method that overcomes the issues inherent in the current approaches was proposed and described. In its development and implementation, the previously described methods for feature extraction, matching, correspondence ranking and transformation estimation were employed. In the first step, an improved contour-based corner detection was used as the feature extraction technique. This was followed by applying the feature matching approach using the proposed dissimilarity metric. In order to improve the performance and accuracy, the correspondence ranking using line features was employed. Finally, in the last step, the best correspondence points were utilized to estimate the homogeneous parameters. This approach enabled the inverse transformation to be calculated, in order to transform the target pixel coordinates and form the image registration application. The proposed framework is not only robust and efficient but can also be generalized for use in similar applications including spatial resolution differences, as well as incorporated into different types of image registration applications. However, it may fail under high degrees of changes and modalities, and in cases where the difference in frequencies or wavelengths is significant.

## Supporting information

S1 Dataset Geometric feature descriptor image registration dataset (GFDRD). (ZIP)
